# Use of improved memory type control charts for monitoring cancer patients recovery time censored data

**DOI:** 10.1038/s41598-024-55731-0

**Published:** 2024-03-07

**Authors:** Syed Muhammad Muslim Raza, Maqbool Hussain Sial, Najam ul Hassan, Getachew Tekle Mekiso, Yusra A. Tashkandy, M. E. Bakr, Anoop Kumar

**Affiliations:** 1 Department of Economics and Statistics, Dr Hasan Murad School of Management, University of Management and Technology, Lahore, Pakistan; 2 Department of Statistics, Virtual University of Pakistan, Lahore, Pakistan; 3 Department of Economics, Thal University, Bhakkar, Pakistan; 4 Department of Statistics, Wachemo University, Hosaino, Ethiopia; 5 Department of Statistics and Operations Research, College of Science, King Saud University, P.O. Box 2455, 11451 Riyadh, Saudi Arabia; 6 Department of Statistics, Faculty of Applied Sciences, Amity University Uttar Pradesh, Lucknow, 226028 India

**Keywords:** CDEC, Average run length (ARL), Cumulative sum, Control charts, Conditional expected values, Conditional median, Type-I censoring, Cancer, Diseases, Health care, Energy science and technology, Mathematics and computing

## Abstract

Control charts are a statistical approach for monitoring cancer data that can assist discover patterns, trends, and unusual deviations in cancer-related data across time. To detect deviations from predicted patterns, control charts are extensively used in quality control and process management. Control charts may be used to track numerous parameters in cancer data, such as incidence rates, death rates, survival time, recovery time, and other related indicators. In this study, CDEC chart is proposed to monitor the cancer patients recovery time censored data. This paper presents a composite dual exponentially weighted moving average Cumulative sum (CDEC) control chart for monitoring cancer patients recovery time censored data. This approach seeks to detect changes in the mean recovery time of cancer patients which usually follows Weibull lifetimes. The results are calculated using type I censored data under known and estimated parameter conditions. We combine the conditional expected value (CEV) and conditional median (CM) approaches, which are extensively used in statistical analysis to determine the central tendency of a dataset, to create an efficient control chart. The suggested chart's performance is assessed using the average run length (ARL), which evaluates how efficiently the chart can detect a change in the process mean. The CDEC chart is compared to existing control charts. A simulation study and a real-world data set related to cancer patients recovery time censored data is used for results illustration. The proposed CDEC control chart is developed for the data monitoring when complete information about the patients are not available. So, instead of doping the patients information we can used the proposed chart to monitor the patients information even if it is censored. The authors conclude that the suggested CDEC chart is more efficient than competitor control charts for monitoring cancer patients recovery time censored data. Overall, this study introduces an efficient new approach for cancer patients recovery time censored data, which might have significant effect on quality control and process improvement across a wide range of healthcare and medical studies.

## Introduction

Control charts are powerful tools used in statistical process control (SPC) to monitor and analyze processes over time. They help organizations ensure that their processes are stable, predictable, and within specified limits. Control charts enable identifying variations and trends that may indicate potential issues or improvements in a process. Control charts have numerous applications in various industries, and they play a crucial role in quality management and process improvement.

Control charts are also applied in the service industry to monitor service delivery metrics, customer satisfaction scores, and process efficiency. Control charts are used in software development to monitor defects, code reviews, and development cycle times, helping teams improve software quality and delivery. Control charts are employed in environmental monitoring to track air and water quality, pollution levels, and other environmental parameters.

The SPC charts can be used to monitor and improve supply chain processes, such as inventory management, order fulfillment, and delivery times.The control charts allow organizations to continuously monitor a process to ensure it is operating within acceptable limits. They help distinguish between common cause variation (inherent in the process) and special cause variation (due to external factors or specific events).

When using control charts, it is essential to gather sufficient and representative data and to follow proper sampling procedures to ensure the reliability and effectiveness of the analysis. Additionally, control charts are not a standalone solution but are part of a broader statistical process control system that includes data collection, analysis, and process improvement methodologies.

Control charts are extensively used in manufacturing to monitor and control production processes, ensuring consistent product quality and minimizing defects. Control charts are applied in healthcare to monitor patient outcomes, track medical errors, and identify opportunities for improvement in healthcare processes.

Cancer is a condition in which some cells in the body develop uncontrolled and spread to other regions of the body.Cancer can begin practically in anyplace in the human body, which contains billions of cells. Human cells normally develop and multiply (a process known as cell division) to generate new cells when the body requires them.

Cells die as they get old or injured, and new cells replace them. When this ordered mechanism fails, aberrant or damaged cells grow and reproduce when they should not. These cells can combine to produce tumour. Tumour can be cancerous or benign (not cancerous).

**Figure 1:**
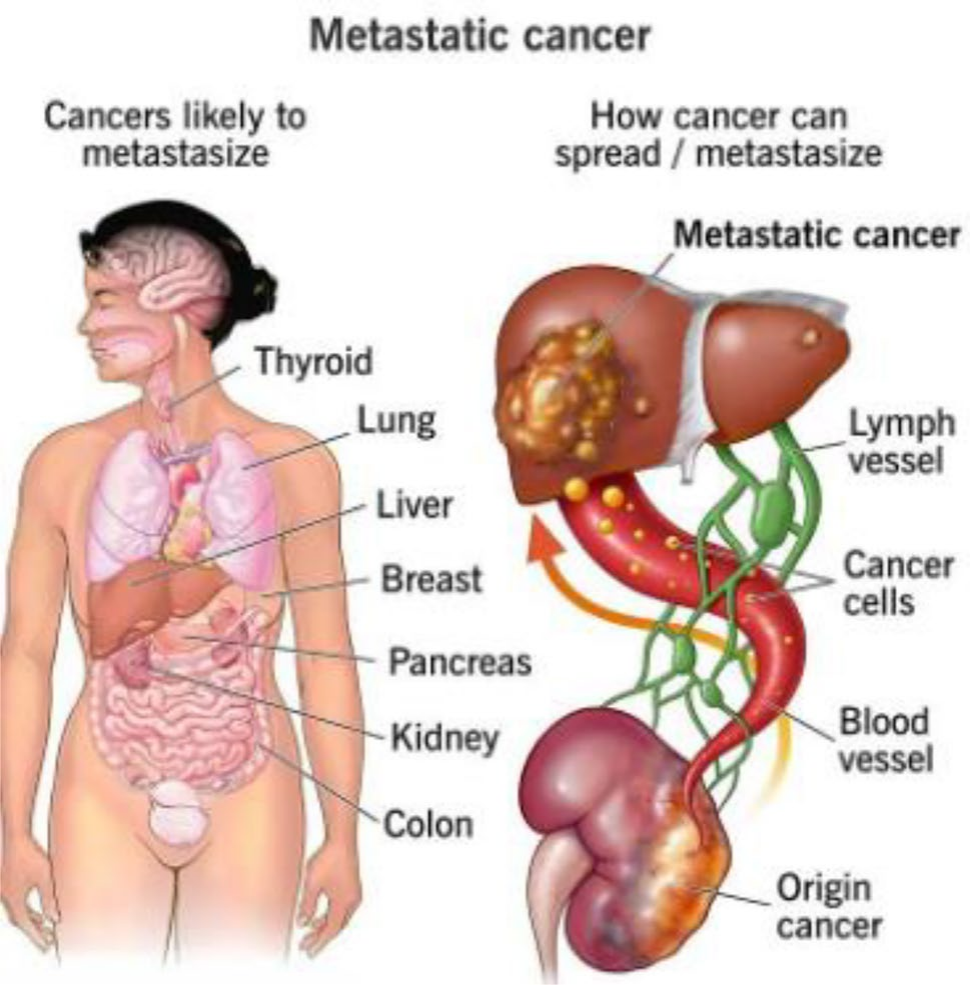
Metastatic Cancer illustration^1^.

Metastatic cancer refers to cancer that has spread beyond the point of origin to other, distant areas of the body (cf. Fig. 1^1^).

Figure 1^1^ illustrate the Metastatic Cancer in distant area of human body. Metastatic cancer, also known as stage IV cancer, is a type of cancer that has spread from its original (primary) site to other parts of the body. In metastasis, cancer cells break away from the primary tumor, enter the bloodstream or lymphatic system, and form new tumors in other organs or tissues. These secondary tumors are called metastases. The spread of cancer cells to distant parts of the body is a complex process that involves several steps. Cancer cells may invade nearby tissues, enter blood vessels or lymphatic channels, and then travel to other parts of the body. Once they reach a new location, these cells can start to grow and form new tumors. The Precision oncology, also known as personalized or targeted therapy, is an approach to cancer treatment that takes into account the specific genetic and molecular characteristics of an individual's cancer. The goal of precision oncology is to tailor treatment strategies to the unique features of each patient's cancer, optimizing the likelihood of a positive response while minimizing side effects^2^. The laboratory turnaround time (TAT) is an important performance metric for laboratories. They use statistical process control (SPC) approaches like CUSUM and EWMA in their study to evaluate a precision medicine project in order to analyse the learning curves of NGS and bio-informatics processes.

^3^ evaluate the efficacy of SGRT in clinical applications using statistical process control (SPC). He used EWMA control charts to analyze outlier fractions and small process shifts from the process mean. By using the control charts and process capability indices derived from this process, he analyze the patient positioning-related OSI performance for each patient.

Censored data is important in many fields, particularly in reliability engineering and medical research, where it is common to observe data that are either partially or completely censored. Censoring occurs when the actual value of an observation is not known, either because the measurement is limited or because the observation is still ongoing at the end of the study period. Censored data can occur in various forms, such as Type-I censoring, Type-II censoring, interval censoring, and progressive censoring. The type of censoring affects the type of statistical analysis that can be used to analyze the data. Ignoring censored data or simply treating it as missing data can result in biased estimates and incorrect conclusions. Therefore, it is important to develop statistical methods that can handle censored data appropriately. Control charts, as discussed earlier, are one such method that can be used to monitor censored data. By accounting for censored data, researchers can obtain more accurate estimates of parameters and improve their understanding of the underlying phenomenon being studied. This can lead to better decision-making and improved product or process design, which ultimately can have a significant impact on various industries and fields, including manufacturing, healthcare, Medical, finance, and engineering.

Identifying assignable causes in lifetime data, particularly in medical and industrial research, is a complex and captivating undertaking. Nevertheless, due to constraints in time and expense, the available failure-time information is often incomplete, referred to as filtered data. The classic control charts, such as Shewhart charts, have significantly lower effectiveness compared to censored control charting based on CEV when it comes to monitoring experiments for potential identification of assignable causes for process improvement.^4,5^ Introduced novel control charting approaches for handling comprehensive data. Conventional charts typically exhibit delayed responsiveness and, as a result, have limited ability to effectively monitor suppressed data. To address the unfavourable characteristics of the monitoring techniques for censored data, a one-sided chart utilizing the conditional expected value (CEV) was devised. The authors conducted an actual research and demonstrated that the concept enables quick identification of process deterioration for monitoring data with high levels of censorship. The CEV technique was employed by^6^ to introduce Shewhart control charts, which were subsequently proven effective for highly censored data in industrial and medical contexts.^7^ suggested the use of lower and upper-sided exponentially weighted moving average (EWMA) control charts, employing the constant exponential variance (CEV) technique, to identify changes in the average value of Weibull quality attributes. In a similar manner,^8,9^ presented CEV EWMA charts for the purpose of monitoring type-I censored data. These charts were designed specifically for the gamma and Gompertz distributions. Following the CEV approach^10,11^ also introduced control charts to monitor type-I censored data. It is to be noted that most of the lifetime distributions are skewed and hence CEV approach may not be appropriate. Thus, contrary to the existing approaches,^10^ proposed a conditional median (CM) based Shewhart chart. The authors^12–17^ proved in a simulated study that their hybrid EWMA control chart with repeated sampling outperforms CEV charts. While monitoring systems for type-II censored data have been published in the literature, this work concentrates on type-I censoring, which is routinely utilized in industry. It is critical to distinguish between the CEV, CM, and imputation techniques. All three techniques are methodologically identical in that they substitute censored or missing data to enhance estimate. The conditional mean and conditional median are used to replace censored observations in the CEV and CM, respectively, while imputation methods are used to replace missing observations in the data. The recent research in the field of data monitoring with efficient control charts includes^18–23^.

The CEV and CM based Composite Dual Exponentially weighted moving average Cumulative sum (CDEC) chart, which has not been discussed before in the literature, is introduced in this study to evaluate cancer patients recovery time censored data. This research focuses on type-I censored data and monitoring the mean level of the Weibull distribution, which was chosen for its flexibility and practical importance in medical/healthcare system. The proposed approach, however, may be extended to other acceptable life expectancy distributions. The novel charts outperform conventional CEV and CM-based EWMA charts in finding assignable causes. The research also looks at how the CDEC chart performs when the scale parameter is determined using the maximum likelihood estimation approach. The average run length is used to assess the efficiency of the suggested charts.

Furthermore, a comparison of censored CM and CEV based existing charts to the censored CDEC charts is also given in this article. The proposed CDEC control chart is developed for the data monitoring when complete information about the patients are not available. So, instead of doping the patients information we can used the proposed chart to monitor the patients information even if it is censored.

The proposed CDEC control chart for monitoring censored data of cancer patients recovery times signify a significant evolution in statistical methodologies within the healthcare domain. These refined charts are specifically tailored to enhance the surveillance and analysis of data related to cancer patients recovery times, where certain information may be censored or incomplete. This improvement is crucial for a more nuanced understanding of the patient outcomes and treatment efficacy. The enhancements in these control charts contribute to heightened sensitivity, adaptability, and precision in identifying patterns, anomalies, or trends in datasets with censored observations. Ultimately, this advancement aids healthcare professionals in making more informed decisions regarding cancer patient care and treatment strategies.

The rest of the study is organized as follows: Section 1: "The CEV and CM based composite dual exponentially weighted moving average cumulative sum (CDEC) control charts" presents the derivations of the CEV and the CM. Besides parameter estimation, the design structure of the censored CDEC is also given in Section 1. The performance of the censored CDEC, Dual EWMA (DE), and Composite Dual EWMA (CDE) charts under different censoring rates is discussed in Section 2: "Performance evaluations". Section 3: "Application of CDEC control chart on real life cancer patients recovery time data" presents a dataset on cancer patients recovery time censored data to illustrate the proposed methodology. Final remarks are given in Section 4: "Conclusion".

## The CEV and CM based composite dual exponentially weighted moving average cumulative sum (CDEC) control charts

This section presents the CEV and CM based Composite Dual Exponentially Weighted Moving Average Cumulative Sum (CDEC) charts for monitoring the mean of the Weibull distribution. In this context, the variable of interest Y represents the lifetime of a product, supposed to follow a Weibull distribution. The Weibull distribution is widely employed in reliability analysis, engineering, and medical studies.

The probability density function of a Weibull random variable *Y* is given by:

1 $$f(y,\alpha ,\beta ) = \frac{\beta }{{\alpha^{\beta } }}y^{\beta - 1} \exp \left( { - \left[ {y/\alpha } \right]^{\beta } } \right)\;\;\;{\text{x}} > 0$$

where $\alpha$ is the scale parameter and $\beta$ is the shape parameter, respectively.

Lets denote Y_*i1*_*, Y *_*i2*_* ….. Y *_*ik*_ the actual lifetime while T_*i1*_*, T *_*i2*_* ….. T *_*ik*_*, i* = *1, 2…, n* denote the lifetimes of failed units in a life testing experiment, i.e., obtained after exercising the type-I right censoring mechanism. Here, n denotes the subgroup size, which may be variable depending upon the situation.The *r* is random here while C (censoring time C) are fixed in advance. Then, we compute the censoring rate by *P*_*c*_ = *1-F(Y* = *c;*$\alpha ,\beta$*),* where *F(x;*$\alpha ,\beta$*)* is the cumulative density function of the Weibull distribution, that is, $P(X \le C) = 1 - \exp \left( { - \left[ {C/\alpha } \right]^{\beta } } \right)$. The mean is denoted by $\mu$ and is given as: $\mu = E(y) = \int\limits_{0}^{\infty } {yf(y)dy} = \alpha \Gamma \left( {1 + \frac{1}{\beta }} \right)$.

The CEV for the Weibull distribution is calculated as:

$$CEV = E(Y\left| Y \right. \ge C) = \frac{{\alpha_{0} \Gamma (D_{c} ,1 + 1/\beta_{0} )}}{{\exp ( - D_{c} )}}$$

Similarly, the conditional median $CM = Median[(Y\left| Y \right. \ge C)]$ is derived as:

2 $$\int_{0}^{m} {y^{\beta - 1} \exp \left[ {\left( { - \frac{y}{\alpha }} \right)^{\beta } } \right]dx = \frac{{\alpha^{\beta } \exp ( - D_{c} )}}{2\beta }}$$

The following expression can be derived using algebraic techniques:

3 $$m = \left[ { - \alpha_{0}^{\beta } \ln \left( {\frac{{2 - \exp ( - D_{c} )}}{2}} \right)} \right]^{{1/\beta_{0} }}$$

where ${\text{D}}_{{\text{c}}} = ({\text{C}}/\alpha_{0} )^{{\beta_{0} }}$, lower incomplete gamma function $\Gamma (z,a) = \int_{y = 0}^{z} {z^{a - 1} \exp ( - z)dz}$ and $\alpha_{0}$, $\beta_{0}$ are the stable-process values of $\alpha$ and $\beta$ respectively.

### Estimation of $\alpha$

To estimate the unknown scale parameter, we first write the likelihood function. The MLE under type-I censoring is given as $\hat{\alpha }_{{_{MLE} }} = \frac{1}{r}\left[ {\sum\limits_{i = 1}^{r} {\phi_{i} (X_{i} )^{{\beta_{0} }} + (s - k)C^{{\beta_{0} }} } } \right]^{{\frac{1}{{\beta_{0} }}}}$**,** where *r* represents the censored units per subgroup, *s* represents the sample size, $X_{i}$ (i = 1,2,3,…,s) shows the lifetime from the Weibull distribution^10^.

### CEV and CM based CDEC control charts

To define CDEC chart, assume $\lambda_{1} ,\;\lambda_{2} ,\lambda_{3} \in \left[ {0.0,1.0} \right]$, and construct two new sequences $E_{1} ,E_{2} ,...,$ and $HE_{1} ,HE_{2} ,...,$ as given below (cited from^5^):

4 $${\text{E}}_{{\text{t}}}^{{{\text{CEV}}}} = \lambda_{1} {\overline{\text{G}}}_{{\text{i}}}^{{{\text{CEV}}}} + \left( {1 - \lambda_{1} } \right){\text{E}}_{{{\text{t}} - 1\left( {{\text{CEV}}} \right)}}$$

and

5 $${\text{HE}}_{{\text{t}}}^{{{\text{CEV}}}} = \lambda_{2} {\text{E}}_{{\text{t}}}^{{{\text{CEV}}}} + \left( {1 - \lambda_{2} } \right){\text{HE}}_{{{\text{t}} - 1\,\left( {{\text{CEV}}} \right)}}$$

Then, calculate the following statistic:

6 $${\text{EWC}}_{{{\text{i}}\left( {{\text{CEV}}} \right)}} = {\text{max}}\left\{ {{\text{HE}}_{{\text{t}}}^{{{\text{CEV}}}} ,\;{\text{intial }}\;{\text{value}}} \right\}/{\text{min}}\left\{ {{\text{HE}}_{{\text{t}}}^{{{\text{CEV}}}} ,{\text{intial}}\;{\text{value}}} \right\}$$

To obtain the CEV hybrid DE monitoring statistic, we calculate:

7 $${\text{DWC}}_{{{\text{i}}\left( {{\text{CEV}}} \right)}} = {\text{max}}\left\{ {{\text{DHE}}_{{\text{t}}}^{{{\text{CEV}}}} ,{\text{intial}}\;{\text{value}}} \right\}/{\text{min}}\left\{ {{\text{DHE}}_{{\text{t}}}^{{{\text{CEV}}}} ,\;{\text{intial}}\;{\text{value}}} \right\}$$

where

8 $${\text{DHE}}_{{\text{t}}}^{{{\text{CEV}}}} = \lambda_{3} {\text{HE}}_{{\text{t}}}^{{{\text{CEV}}}} + \left( {1 - \lambda_{3} } \right){\text{DHE}}_{{{\text{t}} - 1\left( {{\text{CEV}}} \right)}}$$

Let $\overline{{DWC_{i} }}$ represent the mean of i-th subgroup of the observations calculated from Eq. (7), then the CEV CDEC statistic in a relative form is defined as follows:

9 $$CDEC_{(cev)i} = \max \{ CDEC_{i - 1} + \overline{{DWC_{i}^{CEV} }} - m_{o} ,\;m_{o} \} /\min \{ CDEC_{i - 1} + \overline{{DWC_{i}^{CEV} }} - m_{o} ,\;m_{o} \} ,\;\;\,i = 1,2,3, \ldots ,\delta$$

Similarly, the *CM C*DEC statistic in a relative form can be defined as follows:

10 $$CDEC_{(cm)i} = \max \{ CDEC_{i - 1} + \overline{{DWC_{i}^{CM} }} - m_{o} ,\;m_{o} \} /\min \{ CDEC_{i - 1} + \overline{{DWC_{i}^{CM} }} - m_{o} ,\;m_{o} \} ,\;\;\;i = 1,2,3, \ldots ,\delta$$

The quantity *m*_*o*_ in Eq. (10) is a barrier and used to increase the sensitivity of the CEV CDEC *c*ontrol chart. Thus, it needs to be chosen carefully and a very natural choice is $m_{o} = \alpha_{0} \;\Gamma \left( {1 + \frac{1}{{\beta_{0} }}} \right)$. However, the starting value $CDEC_{0}^{{}}$ is assumed zero in this study.

The procedure to determine the control limits and ARLs for the pre-fixed values of P_c_, *m*_*o*_, $n$ and *ARL*_*o*_ are adapted from the study^5^. We have used 10,000 MC Simulations for the evaluation of results.

## Performance evaluations

The efficiency of the CEV and CM CDEC charts is discussed in this section. Besides this, a comparison of CDEC charts to the CEV and CM based CDE charts is also given in this section.

To investigate the efficiency of the charts, the Monte Carlo simulation approach is used to calculate the ARL. The ARL assessment of the CEV and CM CDEC charts is discussed assuming known and estimated scale parameter cases while keeping fixed the shape parameter. For this purpose, in Table 1; we have computed the $UCL_{CDEC(CEV)}$ and $UCL_{CDEC(CM)}$ for subgroups of sizes 3 and 7, *ARL*_*0*_ = *100*_*,*_ $\alpha = 1,\;\beta = 0.5$ and assuming different censoring rates, respectively. It is clear that for a small censoring rate the value of UCL with the estimated parameter is smaller as compared to the fixed scale parameter case, and vice versa. This implies that estimation has a very significant impact on the chart performance because it produces more out-of Control alarms as compared to the known parameter case.

**Table 1:** $$UCL_{CM - CDEC}$$ Values ($$\beta = 0.5$$).

$P_{c}$	*n* = *3, *$\lambda_{1} = \lambda_{2} = 0.2,\;\lambda_{3} = 0.3$		*n* = *7*, $\lambda_{1} = \lambda_{2} = 0.2,\;\lambda_{3} = 0.3$	
	Known parameter	MLE	Known parameter	MLE
0.2	1.45	1.15	1.11	1.09
0.3	1.56	1.68	1.34	1.35
0.5	1.00	1.89	1.22	1.10
0.6	1.47	1.44	1.37	1.96

To calculate the Average Run Length (ARL_1_), we created data using a modified parameter from the Weibull distribution. We then plotted this data against the control limit calculated in Step 3. Next, Steps 4–5 are iterated to compute the mean of subgroups that lie outside the upper control limit (UCL), denoted as ARL_1_. It is important to mention that in certain scenarios, no subgroup monitoring statistic can exceed the Upper Control Limit (UCL). To resolve this issue, merely ignore the iteration at that specific index.

### Effect of estimation

From Table 2, one can notice that for a 30% increase in shift with censoring rate 30%, the ARL_1_ values for CMDE chart is 10.21 and for CM CDE chart is 9.08 and CM CDEC chart is 7.25. A similar pattern is observed for other censoring rates and shifts (increase/decrease). Therefore, it is safe to conclude that the proposed CM CDEC chart outperforms the CM DE chart.

**Table 2:** Out-of Control ARL values for CM CDE, CM-DE and CDEC control chart sequences *for* *n* = 7 and $ARL_{0} = 100,\;\alpha = 0.5,\;\beta = 0.5,\;\lambda_{1} = \lambda_{2} = 0.2,\;\lambda_{3} = 0.3.$

*n*	3											
P_c_	CM DE Chart				CM CDE Chart				CM CDEC Chart			
	Shifts ((+) increase, (−) decrease)				Shifts ((+) increase, (−) decrease)				Shifts ((+) increase, (−) decrease)			
	30% (+)	30% (−)	20% (+)	20% (−)	30% (+)	30% (−)	20% (+)	20% (−)	30% (+)	30% (−)	20% (+)	20% (−)
0.2	4.88	3.62	9.56	12.44	6.16	3.62	7.82	9.67	4.57	1.71	7.58	9.65
0.3	10.21	1.21	19.79	9.45	9.08	1	16.61	9.59	7.25	1.00	15.13	8.81
0.4	17.63	1	43.71	6.18	17.00	1	42.17	5.64	15.54	1.00	41.48	2.82

A similar pattern to Table 2 is observed in Table 3. In comparison to Table 2 Table 3 values of ARL_1_ are higher that Table 3 value so we conclude that ARL_1_ values are smaller for known parameters cases (Table 2 results) than the estimated parameters(Table 3 results) . This comparison also reveals that the impact of estimation on the CM CDEC chart is as significant as it is noticed in the literature.

**Table 3:** Out-of control ARL values for CM CDE, CM-DE and CM CDEC control chart sequences *for n* = *7 with MLE:*$$\overset{\lower0.5em\hbox{$\smash{\scriptscriptstyle\frown}$}}{\alpha } = 0.475,\;\beta = 0.5,\;ARL_{0} = 100,\;\lambda_{1} = \lambda_{2} = 0.2,\;\lambda_{3} = 0.3.$$

*n*	3											
P_c_	CM DE Chart ((+) increase, (−) decrease)				CM CDE Chart ((+) increase, (−) decrease)				CM CDEC Chart ((+) increase, (−) decrease)			
	Shifts				Shifts				Shifts			
	30% (+)	30% (−)	20% (+)	20% (−)	30% (+)	30% (−)	20% (+)	20% (−)	30% (+)	30% (−)	20% (+)	20% (−)
0.2	5.18	4.00	9.70	13.41	7.11	4.12	8.18	9.72	4.83	2.38	7.91	9.77
0.3	10.34	1.58	20.68	10.24	9.39	1	16.89	10.47	7.91	1.83	15.89	9.20
0.4	18.38	1	44.70	7.14	17.48	1	42.47	5.87	15.73	1.89	42.73	3.12

Table 4 shows the comparison of the CM-DE CHART, CM CDE CHART and CM CDEC control charts. The CM CDEC control chart out performs the charts in comparison. From Tables 2 and 4 we can see that the control chart performance for n = 7 is better than n = 3. which shows that the efficiency of the control charts increases as the sample size increases.

**Table 4:** Out-of control ARL values for CM CDE, CM-DE and CDEC control chart sequences *for n* = *3 and *$ARL_{0} = 100,\;\alpha = 0.5,\;\beta = 0.5,\;\lambda_{1} = \lambda_{2} = 0.2,\;\lambda_{3} = 0.3.$

*n*	3											
P_c_	CM-DE Chart				CM CDE Chart				CM CDEC Chart			
	Shifts ((+) increase, (−) decrease)				Shifts ((+) increase, (−) decrease)				Shifts ((+) increase, (−) decrease)			
	30% (+)	30% (−)	20% (+)	20% (−)	30% (+)	30% (−)	20% (+)	20% (−)	30% (+)	30% (−)	20% (+)	20% (−)
0.2	7.40	5.70	11.70	14.20	7.31	3.76	10.28	11.20	6.37	2.56	8.54	11.03
0.3	11.75	3.49	20.57	10.71	9.43	2.24	16.98	11.64	9.24	2.58	17.12	10.08
0.4	20.34	3.76	44.52	6.80	17.79	1.62	44.25	8.09	15.74	1.82	42.68	4.54

## Application of CDEC control chart on real life cancer patients recovery time data

### Real life cancer patients recovery time data

The data was collected using a two-stage sampling method. In the first stage, we selected Lahore District from all other districts of Punjab. In Lahore, there are several cancer research centers such as Shaukat Khanum Memorial Cancer Hospital and Research Centre, Anmol, etc. In the second stage, we selected the Shaukat Khanum Memorial Cancer Hospital and Research Centre. Now, using Yamane’s method with a confidence level of 90%, P = 0.2, and a precision of 10%, we determined the sample size (n = 45) from the research center. The sample was selected using consecutive sampling during the period 2021–2023. The database of Shaukat Khanum Memorial Cancer Hospital and Research Centre was used to initially select the sample of 35 CHLs and 10 NLPHLs diagnosed either on Trucut biopsy or excision biopsy. A telephonic survey was conducted to collect the recovery time from each selected patient included in the sample.

Some patients' recovery time was not reported due to unavailability during the survey response collection. Therefore, their information is considered as type I censored.

**Figure 2:**
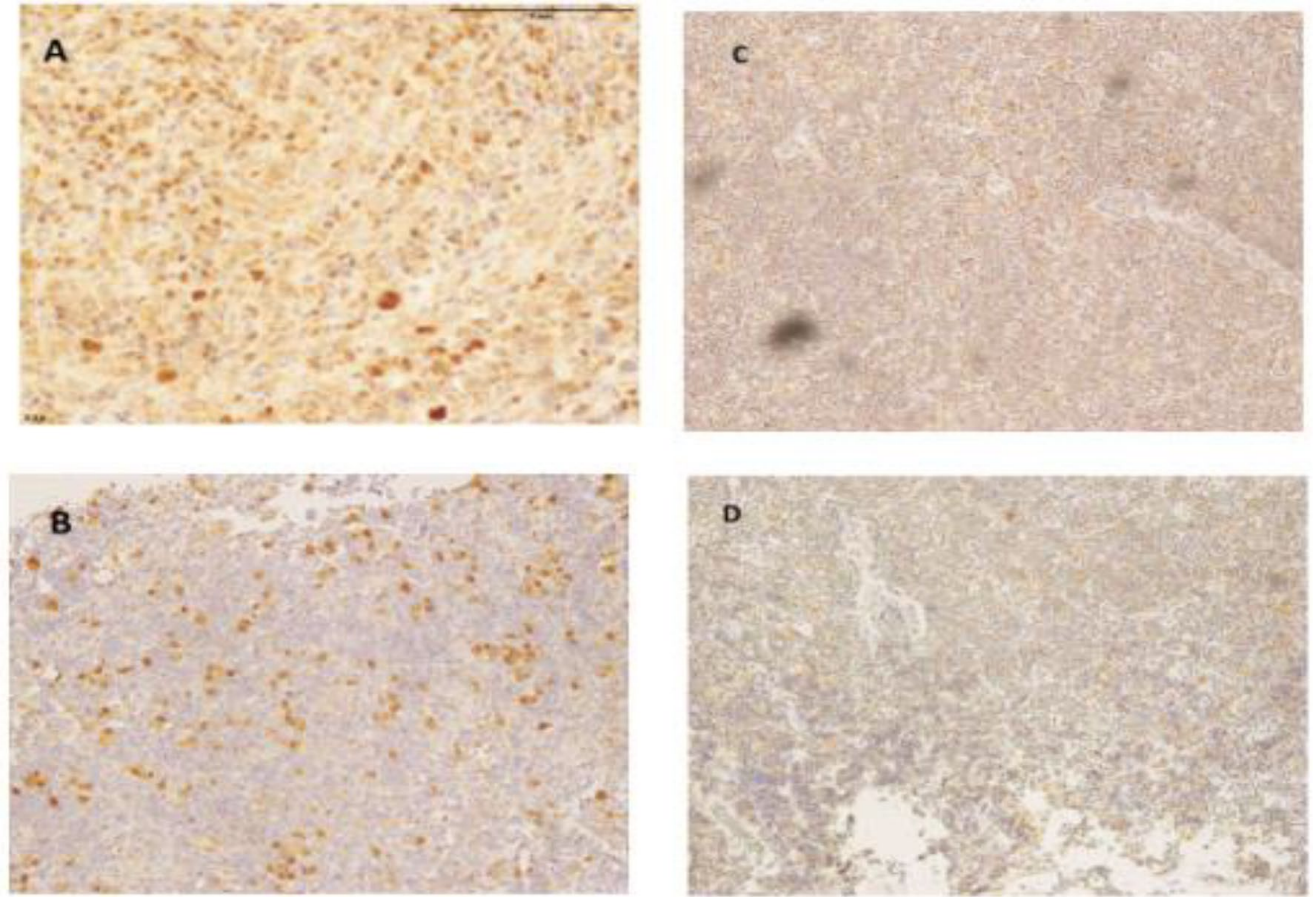
(**A**), GATA3 staining in CHL. (**B**) STAT6 staining in CHL. Images (A,B) demonstrate Reed Sternberg cells which are positive for GATA3 and STAT6 positive. (**C**) GATA3 staining in NLPHL. (**D**) STAT6 staining in NLPHL. Images(**C**,**D**) demonstrate LP cells which are negative for GATA3 and STAT6.

It is observed that all the cases of NLPHL were negative for GATA3, 80% cases showed no staining and 20% cases showed only cytoplasmic blush (Fig. 2). The patient recovery time (in years) is recorded from the patients.

### Application of CDEC chart

The proposed control chart can be used for the monitoring of cancer patient’s recovery time data especially when the complete information may not be available. The patient’s recovery time data may consists of partial information and some of the respondent’s recovery time is unknown due to non-availability of respondents. The unknown recovery time is treated as type I censored data. The data is collected from the patients suffering from Stage I and II cancer. The data available on cancer patients (of Stage I and II) recovery time from the Shaukat Khanum Memorial cancer hospital and research cancer is used for the development of proposed control chart (in Phase I monitoring).

**Figure 3:**
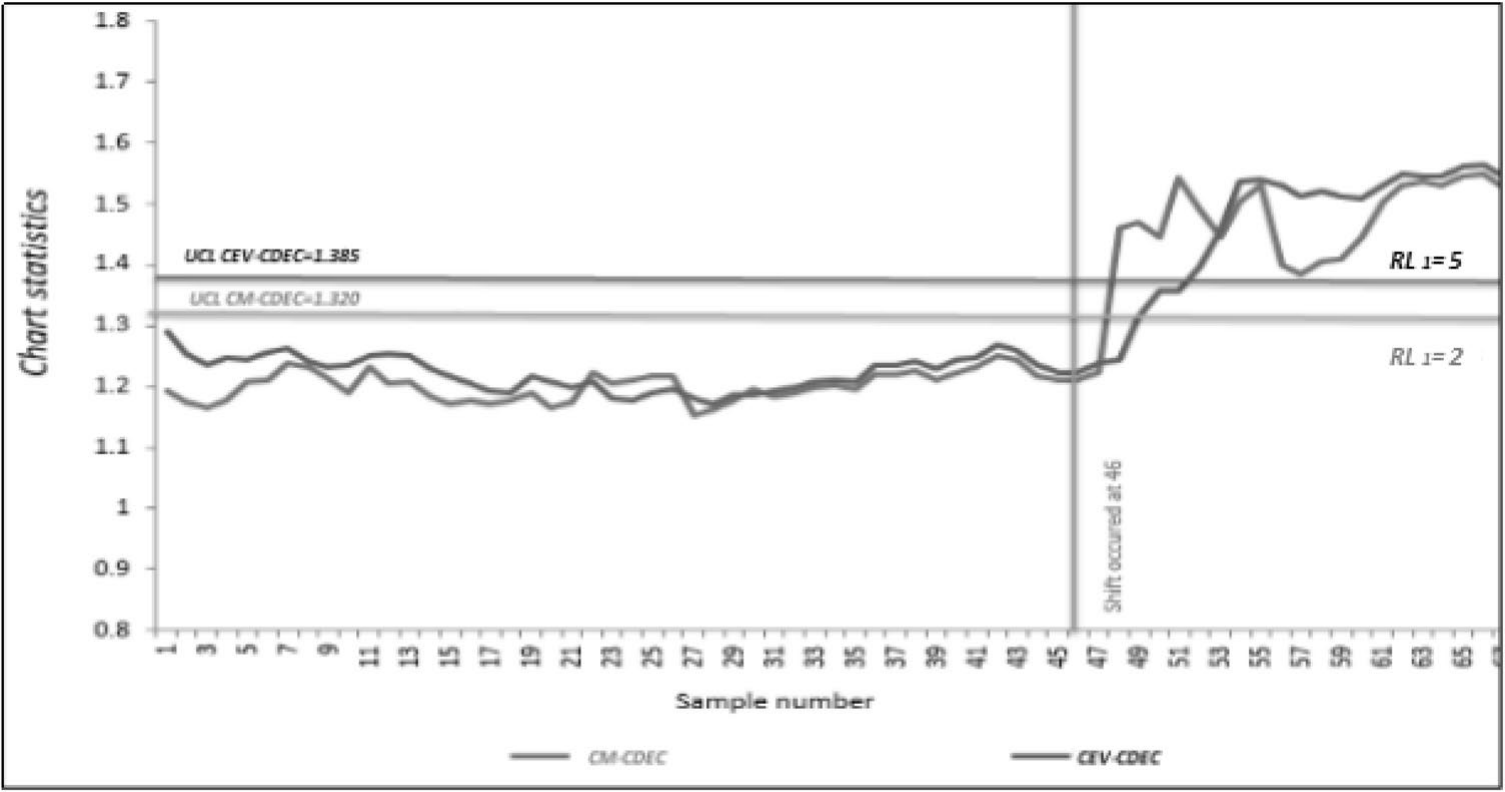
A Comparison of CM CDEC and CEV CDEC charts using 25% increase in the mean for the recovery time.

The distribution of the real life data is checked by Easy fit software. The distribution is found as Weibull distribution with scale parameter $\alpha$ = 0.1 and the shape parameter $\beta$ = 0.5. For phase II monitoring the 25% increase in shift is introduced in the scale parameter, keeping the shape parameter fixed. The data is monitored through existing control charts^5^ and new proposed CDEC control charts. A 20% censoring rate is assumed. Based on these assumptions, the proposed hybrid control chart is developed. From Fig. 3, it is observed that the CM and CEV CDEC control charts do not raise any out-of Control signal till the 46th sampled patient recovery time. Hence, to assess the superiority of the proposal, a data set consisting of 20 observations is generated, i.e., after the 46th respondent data. To generate the shifted data, a 25% increasing shift in the mean recovery time is introduced using the Weibull distribution. The censoring time is 1.1 years while In-Control Run Length (RL_0_) = 46. The CM value for the above-mentioned specifications is 1.1. For the shifted samples, the CM CDEC produced an out-of Control signal at the 2nd sample while the CEV CDEC control chart at the 5th sample (cf. Figure 3). Thus, the CM CDEC chart is more efficient to detect an assignable cause than the CEV CDEC control chart.

The proposed control chart is quite useful in monitoring the suppressed data. In real life, especially in medical trials, we face the problem when complete data from patients is not accessible during their recovery period. Monitoring such data using the proposed charting approach helps in both monitoring the data and spotting unusual patterns in the data.

In the real life; the Phase II monitoring occurs when the patient’s recovery time is increased. The recovery time for cancer patients can vary widely depending on several factors, including the type of cancer, stage at diagnosis, overall health of the patient, and the treatment approach. Here are some reasons why the recovery time for cancer patients may increase:

#### Type and stage of cancer

Different types of cancer have varying rates of growth and aggressiveness. Similarly, the stage at which cancer is diagnosed plays a crucial role. In general, cancers detected at an early stage may have better outcomes and shorter recovery times than those diagnosed at advanced stages.

#### Treatment modalities

The type of treatment a patient receives can significantly impact recovery time. Surgery, chemotherapy, radiation therapy, immuno-therapy, and targeted therapies are common cancer treatments, and each has its own set of side effects and recovery periods. Some treatments may require more time for the body to recover and heal.

#### Individual health status

The overall health and fitness of the patient before cancer diagnosis can affect recovery time. Patients with underlying health conditions or weakened immune systems may take longer to recover from cancer treatments.

#### Adverse effects of treatment

Cancer treatments often come with side effects such as fatigue, nausea, pain, and immune system suppression. These side effects can extend the recovery time as the body needs time to recover from the impact of treatment.

#### Complications

Sometimes, unexpected complications can arise during or after treatment, leading to a longer recovery period. These complications may include infections, surgical complications, or adverse reactions to medications.

#### Psychological and emotional factors

Cancer and its treatment can have a profound impact on a patient's mental health. Emotional stress, anxiety, and depression can affect the overall well-being and potentially slow down the recovery process.

#### Support system

The level of support a patient receives from family, friends, and healthcare professionals can influence recovery. Adequate support can positively impact mental and emotional well-being, which, in turn, may contribute to a smoother recovery.

#### Follow-up care

Ongoing monitoring and follow-up care are crucial for cancer survivors. Regular check-ups and screenings are necessary to detect any signs of recurrence or complications early on.

We have applied the existing CM-DE, CM CDE and proposed CM CDEC control charts for monitoring the real life cancer patients recovery time data (of Shaukat Khanum Memorial cancer hospital and research Centre). Table 5 shows that for a 25% increase in shift with censoring rate 20%, the RL_1_ values for CM-DE chart is 9; CM CDE chart is 7 and CM CDEC chart is 2. Therefore, it is safe to conclude that the proposed CM CDEC chart outperforms the previously existing (CM-DE and CM CDE) control charts.

**Table 5:** Out-of Control Run Length (RL_1_) values for CM CDE, CM-DE and CDEC control chart using real life cancer patients recovery time data (of Shaukat Khanum Memorial cancer hospital and research Centre) $(\alpha = 1.2,\;\beta = 0.5,\;\lambda_{1} = \lambda_{2} = 0.2,\;\lambda_{3} = 0.3,\;25\% \;Increase\;in\;shift)$.

P_c =_0.2	CM-DE Chart (RL_1_)	CM CDE Chart (RL_1_)	CM CDEC Chart (RL_1_)
	9	7	2

Table 6 shows the simulated samples and calculation for CEV CDEC control charting parameters *for n* = *7****.***

**Table 6:** Samples and calculation for CEV CDEC control charting parameters *for n* = *7.*

Samples							Charting statistics	UCL	LCL	CL	SD BAR
5.675529	5.10485	5.250828	5.343735667	5.509632333	5.250828	5.343735667	5.304528	6.980597	3.010091	4.99018264	0.734534
4.306671	3.911458	5.974296	4.730808333	4.518739667	5.974296	4.730808333	4.553507	6.980597	3.010091	4.99018264	0.734534
5.811056	4.351492	5.580828	5.247792	5.529424	5.580828	5.247792	5.386179	6.980597	3.010091	4.99018264	0.734534
3.765854	5.837349	5.943186	5.182129667	4.473991833	5.943186	5.182129667	5.44782	6.980597	3.010091	4.99018264	0.734534
4.856204	6.08718	3.066713	4.670032333	4.763118167	3.066713	4.670032333	4.749646	6.980597	3.010091	4.99018264	0.734534
2.25401	4.541966	3.959618	3.585198	2.919604	3.959618	3.585198	3.74318	6.980597	3.010091	4.99018264	0.734534
3.721541	5.88579	3.704357	4.437229333	4.079385167	3.704357	4.437229333	4.150284	6.980597	3.010091	4.99018264	0.734534
7.142865	4.803882	6.118206	6.021651	6.582258	6.118206	6.021651	6.062991	6.980597	3.010091	4.99018264	0.734534
5.671357	3.11572	4.863337	4.550138	5.1107475	4.863337	4.550138	4.683207	6.980597	3.010091	4.99018264	0.734534
5.68822	6.833511	3.07235	5.198027	5.4431235	3.07235	5.198027	5.406056	6.980597	3.010091	4.99018264	0.734534
5.428717	2.88539	5.094755	4.469620667	4.949168833	5.094755	4.469620667	4.728041	6.980597	3.010091	4.99018264	0.734534
6.021958	4.421367	4.728521	5.057282	5.53962	4.728521	5.057282	4.919843	6.980597	3.010091	4.99018264	0.734534
3.321292	4.616851	3.226516	3.721553	3.5214225	3.226516	3.721553	3.558436	6.980597	3.010091	4.99018264	0.734534
6.365204	5.873771	4.885739	5.708238	6.036721	4.885739	5.708238	5.778671	6.980597	3.010091	4.99018264	0.734534
6.633603	6.01011	4.689272	5.777661667	6.205632333	4.689272	5.777661667	5.876464	6.980597	3.010091	4.99018264	0.734534
3.528413	6.235686	4.596872	4.786990333	4.157701667	4.596872	4.786990333	4.705742	6.980597	3.010091	4.99018264	0.734534
4.880359	3.502575	3.6105	3.997811333	4.439085167	3.6105	3.997811333	3.839579	6.980597	3.010091	4.99018264	0.734534
7.217269	4.975451	3.825802	5.339507333	6.278388167	3.825802	5.339507333	5.184516	6.980597	3.010091	4.99018264	0.734534
4.717599	6.416565	3.981648	5.038604	4.8781015	3.981648	5.038604	4.902407	6.980597	3.010091	4.99018264	0.734534
2.920784	4.282614	3.016035	3.406477667	3.163630833	3.016035	3.406477667	3.247292	6.980597	3.010091	4.99018264	0.734534
4.449197	5.709388	5.451159	5.203248	4.8262225	5.451159	5.203248	5.307106	6.980597	3.010091	4.99018264	0.734534
7.050713	3.364944	5.103587	5.173081333	6.111897167	5.103587	5.173081333	5.143304	6.980597	3.010091	4.99018264	0.734534
4.153359	4.565328	5.283764	4.667483667	4.410421333	5.283764	4.667483667	4.623908	6.980597	3.010091	4.99018264	0.734534
5.191911	4.482289	5.07797	4.91739	5.0546505	5.07797	4.91739	4.984151	6.980597	3.010091	4.99018264	0.734534
3.806668	4.020909	5.541407	4.456328	4.131498	5.541407	4.456328	4.276625	6.980597	3.010091	4.99018264	0.734534
5.91171	6.822834	4.899645	5.878063	5.8948865	4.899645	5.878063	5.89248	6.980597	3.010091	4.99018264	0.734534
4.763376	2.985271	3.383203	3.710616667	4.236996333	3.383203	3.710616667	3.573055	6.980597	3.010091	4.99018264	0.734534
5.828828	4.141981	5.292341	5.087716667	5.458272333	5.292341	5.087716667	5.174671	6.980597	3.010091	4.99018264	0.734534
4.823521	5.852225	5.693756	5.456500667	5.140010833	5.693756	5.456500667	5.555018	6.980597	3.010091	4.99018264	0.734534
4.901828	4.245094	4.67986	4.608927333	4.755377667	4.67986	4.608927333	4.639124	6.980597	3.010091	4.99018264	0.734534
6.534007	4.337895	6.423141	5.765014333	6.149510667	6.423141	5.765014333	6.032015	6.980597	3.010091	4.99018264	0.734534
6.624266	3.889244	5.390714	5.301408	5.962837	5.390714	5.301408	5.339658	6.980597	3.010091	4.99018264	0.734534
5.845819	5.827471	4.531777	5.401689	5.623754	4.531777	5.401689	5.572703	6.980597	3.010091	4.99018264	0.734534
5.474306	4.245077	3.312901	4.344094667	4.909200333	3.312901	4.344094667	4.30171	6.980597	3.010091	4.99018264	0.734534
4.388281	4.210381	5.562507	4.720389667	4.554335333	5.562507	4.720389667	4.583094	6.980597	3.010091	4.99018264	0.734534
5.429981	4.276572	4.269004	4.658519	5.04425	4.269004	4.658519	4.505443	6.980597	3.010091	4.99018264	0.734534
4.182783	5.702815	4.985842	4.957146667	4.569964833	4.985842	4.957146667	4.969442	6.980597	3.010091	4.99018264	0.734534
6.191727	3.32326	2.309483	3.94149	5.0666085	2.309483	3.94149	3.680416	6.980597	3.010091	4.99018264	0.734534
7.340281	6.166309	2.666247	5.390945667	6.365613333	2.666247	5.390945667	5.717956	6.980597	3.010091	4.99018264	0.734534
4.783173	6.046561	5.281741	5.370491667	5.076832333	5.281741	5.370491667	5.332563	6.980597	3.010091	4.99018264	0.734534
4.424859	4.689727	4.578742	4.564442667	4.494650833	4.578742	4.564442667	4.570561	6.980597	3.010091	4.99018264	0.734534
7.749881	6.008586	4.253907	6.004124667	6.877002833	4.253907	6.004124667	6.006036	6.980597	3.010091	4.99018264	0.734534
7.411178	6.683396	4.334621	6.143065	6.7771215	4.334621	6.143065	6.370499	6.980597	3.010091	4.99018264	0.734534
7.725089	6.209489	4.06349	5.999356	6.8622225	4.06349	5.999356	6.089243	6.980597	3.010091	4.99018264	0.734534
4.098049	6.063624	3.713837	4.62517	4.3616095	3.713837	4.62517	4.4059	6.980597	3.010091	4.99018264	0.734534
5.638004	4.938797	5.641523	5.406108	5.522056	5.641523	5.406108	5.499005	6.980597	3.010091	4.99018264	0.734534
2.261085	7.56727	4.174903	4.667752667	3.464418833	4.174903	4.667752667	4.457576	6.980597	3.010091	4.99018264	0.734534
4.739951	5.920966	4.13286	4.931259	4.835605	4.13286	4.931259	4.849809	6.980597	3.010091	4.99018264	0.734534
4.48649	5.783055	4.679288	4.982944333	4.734717167	4.679288	4.982944333	4.856983	6.980597	3.010091	4.99018264	0.734534
5.106262	2.87988	4.260907	4.082349667	4.594305833	4.260907	4.082349667	4.158592	6.980597	3.010091	4.99018264	0.734534
5.294058	3.442415	4.944924	4.560465667	4.927261833	4.944924	4.560465667	4.721098	6.980597	3.010091	4.99018264	0.734534
4.540502	4.325219	9.057352	5.974357667	5.257429833	9.057352	5.974357667	5.393408	6.980597	3.010091	4.99018264	0.734534
3.974602	4.790526	4.620093	4.461740333	4.218171167	4.620093	4.461740333	4.528121	6.980597	3.010091	4.99018264	0.734534
5.57129	6.376151	6.997416	6.314952333	5.943121167	6.997416	6.314952333	6.341153	6.980597	3.010091	4.99018264	0.734534
4.099452	2.310166	5.280023	3.896547	3.9979995	5.280023	3.896547	3.983274	6.980597	3.010091	4.99018264	0.734534
3.540908	7.135742	5.236984	5.304544667	4.422726333	5.236984	5.304544667	5.275596	6.980597	3.010091	4.99018264	0.734534
5.862522	5.468016	7.681953	6.337497	6.1000095	7.681953	6.337497	6.139398	6.980597	3.010091	4.99018264	0.734534
3.398433	6.825256	4.511027	4.911572	4.1550025	4.511027	4.911572	4.741258	6.980597	3.010091	4.99018264	0.734534
4.951761	3.419356	4.453616	4.274911	4.613336	4.453616	4.274911	4.3509	6.980597	3.010091	4.99018264	0.734534
3.429133	3.844412	6.047054	4.440199667	3.934666333	6.047054	4.440199667	4.192591	6.980597	3.010091	4.99018264	0.734534
5.134808	3.881292	4.129734	4.381944667	4.758376333	4.129734	4.381944667	4.276399	6.980597	3.010091	4.99018264	0.734534
4.288021	3.249454	3.223976	3.587150333	3.937585667	3.223976	3.587150333	3.45113	6.980597	3.010091	4.99018264	0.734534
5.888044	2.889265	5.429319	4.735542667	5.311793333	5.429319	4.735542667	5.023568	6.980597	3.010091	4.99018264	0.734534
4.788309	5.147643	4.329253	4.755068333	4.771688667	4.329253	4.755068333	4.769301	6.980597	3.010091	4.99018264	0.734534
4.715687	7.177859	4.26067	5.384738667	5.050212833	4.26067	5.384738667	5.106819	6.980597	3.010091	4.99018264	0.734534
4.624745	7.194517	5.131934	5.650398667	5.137571833	5.131934	5.650398667	5.43346	6.980597	3.010091	4.99018264	0.734534
6.943164	4.347986	3.497123	4.929424333	5.936294167	3.497123	4.929424333	4.68434	6.980597	3.010091	4.99018264	0.734534
4.98238	5.255177	7.038943	5.758833333	5.370606667	7.038943	5.758833333	5.550573	6.980597	3.010091	4.99018264	0.734534
6.785792	7.407878	5.563556	6.585742	6.685767	5.563556	6.585742	6.670898	6.980597	3.010091	4.99018264	0.734534
3.321292	4.616851	3.226516	3.721553	3.5214225	3.226516	3.721553	3.558436	6.980597	3.010091	4.99018264	0.734534
4.388281	4.210381	5.562507	4.720389667	4.554335333	5.562507	4.720389667	4.583094	6.980597	3.010091	4.99018264	0.734534
5.429981	4.276572	4.269004	4.658519	5.04425	4.269004	4.658519	4.505443	6.980597	3.010091	4.99018264	0.734534
4.182783	5.702815	4.985842	4.957146667	4.569964833	4.985842	4.957146667	4.969442	6.980597	3.010091	4.99018264	0.734534
6.191727	3.32326	2.309483	3.94149	5.0666085	2.309483	3.94149	3.680416	6.980597	3.010091	4.99018264	0.734534
7.340281	6.166309	2.666247	5.390945667	6.365613333	2.666247	5.390945667	5.717956	6.980597	3.010091	4.99018264	0.734534
4.783173	6.046561	5.281741	5.370491667	5.076832333	5.281741	5.370491667	5.332563	6.980597	3.010091	4.99018264	0.734534
4.424859	4.689727	4.578742	4.564442667	4.494650833	4.578742	4.564442667	4.570561	6.980597	3.010091	4.99018264	0.734534
7.749881	6.008586	4.253907	6.004124667	6.877002833	4.253907	6.004124667	6.006036	6.980597	3.010091	4.99018264	0.734534
7.411178	6.683396	4.334621	6.143065	6.7771215	4.334621	6.143065	6.370499	6.980597	3.010091	4.99018264	0.734534
7.725089	6.209489	4.06349	5.999356	6.8622225	4.06349	5.999356	6.089243	6.980597	3.010091	4.99018264	0.734534
4.098049	6.063624	3.713837	4.62517	4.3616095	3.713837	4.62517	4.4059	6.980597	3.010091	4.99018264	0.734534
5.638004	4.938797	5.641523	5.406108	5.522056	5.641523	5.406108	5.499005	6.980597	3.010091	4.99018264	0.734534
2.261085	7.56727	4.174903	4.667752667	3.464418833	4.174903	4.667752667	4.457576	6.980597	3.010091	4.99018264	0.734534
4.739951	5.920966	4.13286	4.931259	4.835605	4.13286	4.931259	4.849809	6.980597	3.010091	4.99018264	0.734534
4.48649	5.783055	4.679288	4.982944333	4.734717167	4.679288	4.982944333	4.856983	6.980597	3.010091	4.99018264	0.734534
5.106262	2.87988	4.260907	4.082349667	4.594305833	4.260907	4.082349667	4.158592	6.980597	3.010091	4.99018264	0.734534
5.294058	3.442415	4.944924	4.560465667	4.927261833	4.944924	4.560465667	4.721098	6.980597	3.010091	4.99018264	0.734534
4.540502	4.325219	9.057352	5.974357667	5.257429833	9.057352	5.974357667	5.393408	6.980597	3.010091	4.99018264	0.734534
3.974602	4.790526	4.620093	4.461740333	4.218171167	4.620093	4.461740333	4.528121	6.980597	3.010091	4.99018264	0.734534
5.57129	6.376151	6.997416	6.314952333	5.943121167	6.997416	6.314952333	6.341153	6.980597	3.010091	4.99018264	0.734534
4.099452	2.310166	5.280023	3.896547	3.9979995	5.280023	3.896547	3.983274	6.980597	3.010091	4.99018264	0.734534
3.540908	7.135742	5.236984	5.304544667	4.422726333	5.236984	5.304544667	5.275596	6.980597	3.010091	4.99018264	0.734534
5.862522	5.468016	7.681953	6.337497	6.1000095	7.681953	6.337497	6.139398	6.980597	3.010091	4.99018264	0.734534
3.398433	6.825256	4.511027	4.911572	4.1550025	4.511027	4.911572	4.741258	6.980597	3.010091	4.99018264	0.734534
4.951761	3.419356	4.453616	4.274911	4.613336	4.453616	4.274911	4.3509	6.980597	3.010091	4.99018264	0.734534
3.429133	3.844412	6.047054	4.440199667	3.934666333	6.047054	4.440199667	4.192591	6.980597	3.010091	4.99018264	0.734534
5.134808	3.881292	4.129734	4.381944667	4.758376333	4.129734	4.381944667	4.276399	6.980597	3.010091	4.99018264	0.734534
4.288021	3.249454	3.223976	3.587150333	3.937585667	3.223976	3.587150333	3.45113	6.980597	3.010091	4.99018264	0.734534
5.888044	2.889265	5.429319	4.735542667	5.311793333	5.429319	4.735542667	5.023568	6.980597	3.010091	4.99018264	0.734534
4.788309	5.147643	4.329253	4.755068333	4.771688667	4.329253	4.755068333	4.769301	6.980597	3.010091	4.99018264	0.734534
4.715687	7.177859	4.26067	5.384738667	5.050212833	4.26067	5.384738667	5.106819	6.980597	3.010091	4.99018264	0.734534
4.624745	7.194517	5.131934	5.650398667	5.137571833	5.131934	5.650398667	5.43346	6.980597	3.010091	4.99018264	0.734534
6.943164	4.347986	3.497123	4.929424333	5.936294167	3.497123	4.929424333	4.68434	6.980597	3.010091	4.99018264	0.734534
4.98238	5.255177	7.038943	5.758833333	5.370606667	7.038943	5.758833333	5.550573	6.980597	3.010091	4.99018264	0.734534
6.785792	7.407878	5.563556	6.585742	6.685767	5.563556	6.585742	6.670898	6.980597	3.010091	4.99018264	0.734534
3.321292	4.616851	3.226516	3.721553	3.5214225	3.226516	3.721553	3.558436	6.980597	3.010091	4.99018264	0.734534
6.191727	3.32326	2.309483	3.94149	5.0666085	2.309483	3.94149	3.680416	6.980597	3.010091	4.99018264	0.734534
7.340281	6.166309	2.666247	5.390945667	6.365613333	2.666247	5.390945667	5.717956	6.980597	3.010091	4.99018264	0.734534
4.783173	6.046561	5.281741	5.370491667	5.076832333	5.281741	5.370491667	5.332563	6.980597	3.010091	4.99018264	0.734534
4.424859	4.689727	4.578742	4.564442667	4.494650833	4.578742	4.564442667	4.570561	6.980597	3.010091	4.99018264	0.734534
7.749881	6.008586	4.253907	6.004124667	6.877002833	4.253907	6.004124667	6.006036	6.980597	3.010091	4.99018264	0.734534
7.411178	6.683396	4.334621	6.143065	6.7771215	4.334621	6.143065	6.370499	6.980597	3.010091	4.99018264	0.734534
7.725089	6.209489	4.06349	5.999356	6.8622225	4.06349	5.999356	6.089243	6.980597	3.010091	4.99018264	0.734534
4.098049	6.063624	3.713837	4.62517	4.3616095	3.713837	4.62517	4.4059	6.980597	3.010091	4.99018264	0.734534
5.638004	4.938797	5.641523	5.406108	5.522056	5.641523	5.406108	5.499005	6.980597	3.010091	4.99018264	0.734534
2.261085	7.56727	4.174903	4.667752667	3.464418833	4.174903	4.667752667	4.457576	6.980597	3.010091	4.99018264	0.734534
4.739951	5.920966	4.13286	4.931259	4.835605	4.13286	4.931259	4.849809	6.980597	3.010091	4.99018264	0.734534
4.48649	5.783055	4.679288	4.982944333	4.734717167	4.679288	4.982944333	4.856983	6.980597	3.010091	4.99018264	0.734534
5.106262	2.87988	4.260907	4.082349667	4.594305833	4.260907	4.082349667	4.158592	6.980597	3.010091	4.99018264	0.734534
5.294058	3.442415	4.944924	4.560465667	4.927261833	4.944924	4.560465667	4.721098	6.980597	3.010091	4.99018264	0.734534
4.540502	4.325219	9.057352	5.974357667	5.257429833	9.057352	5.974357667	5.393408	6.980597	3.010091	4.99018264	0.734534
3.974602	4.790526	4.620093	4.461740333	4.218171167	4.620093	4.461740333	4.528121	6.980597	3.010091	4.99018264	0.734534

Table 7 shows the simulated samples and calculation for CM CDEC control charting parameters *for n* = *7****.***

**Table 7:** Samples and calculation for CM CDEC control charting parameters *for n* = *7.*

Samples							Charting statistics	UCL	LCL	CL	SD BAR
5.675529	5.10485	5.250828	5.343735667	5.509632333	5.250828	5.343735667	4.412505107	4.971233035	5.112390877	5.22078623	4.356900716
4.306671	3.911458	5.974296	4.730808333	4.518739667	5.974296	4.730808333	5.171068015	4.971233035	5.112390877	5.22078623	4.621825203
5.811056	4.351492	5.580828	5.247792	5.529424	5.580828	5.247792	4.362081627	4.971233035	5.112390877	5.22078623	5.033161457
3.765854	5.837349	5.943186	5.182129667	4.473991833	5.943186	5.182129667	4.576885187	4.971233035	5.112390877	5.22078623	4.317598591
4.856204	6.08718	3.066713	4.670032333	4.763118167	3.066713	4.670032333	2.931969675	4.971233035	5.112390877	5.22078623	4.435784419
2.25401	4.541966	3.959618	3.585198	2.919604	3.959618	3.585198	2.534289732	4.971233035	5.112390877	5.22078623	2.817097546
3.721541	5.88579	3.704357	4.437229333	4.079385167	3.704357	4.437229333	3.226430084	4.971233035	5.112390877	5.22078623	3.966517536
7.142865	4.803882	6.118206	6.021651	6.582258	6.118206	6.021651	5.486339064	4.971233035	5.112390877	5.22078623	5.258846603
5.671357	3.11572	4.863337	4.550138	5.1107475	4.863337	4.550138	4.280141411	4.971233035	5.112390877	5.22078623	4.402959286
5.68822	6.833511	3.07235	5.198027	5.4431235	3.07235	5.198027	3.035666251	4.971233035	5.112390877	5.22078623	4.918921605
5.428717	2.88539	5.094755	4.469620667	4.949168833	5.094755	4.469620667	4.926355456	4.971233035	5.112390877	5.22078623	3.624123837
6.021958	4.421367	4.728521	5.057282	5.53962	4.728521	5.057282	3.663970165	4.971233035	5.112390877	5.22078623	5.041004117
3.321292	4.616851	3.226516	3.721553	3.5214225	3.226516	3.721553	3.219187463	4.971233035	5.112390877	5.22078623	3.235808748
6.365204	5.873771	4.885739	5.708238	6.036721	4.885739	5.708238	4.865018865	4.971233035	5.112390877	5.22078623	5.486509176
6.633603	6.01011	4.689272	5.777661667	6.205632333	4.689272	5.777661667	3.711038549	4.971233035	5.112390877	5.22078623	5.319830905
3.528413	6.235686	4.596872	4.786990333	4.157701667	4.596872	4.786990333	4.348966745	4.971233035	5.112390877	5.22078623	3.930385411
4.880359	3.502575	3.6105	3.997811333	4.439085167	3.6105	3.997811333	2.561744348	4.971233035	5.112390877	5.22078623	3.430245619
7.217269	4.975451	3.825802	5.339507333	6.278388167	3.825802	5.339507333	2.548762741	4.971233035	5.112390877	5.22078623	4.433877789
4.717599	6.416565	3.981648	5.038604	4.8781015	3.981648	5.038604	2.649301905	4.971233035	5.112390877	5.22078623	4.205813468
2.920784	4.282614	3.016035	3.406477667	3.163630833	3.016035	3.406477667	2.968800658	4.971233035	5.112390877	5.22078623	3.399565883
4.449197	5.709388	5.451159	5.203248	4.8262225	5.451159	5.203248	4.962904129	4.971233035	5.112390877	5.22078623	4.82561023
7.050713	3.364944	5.103587	5.173081333	6.111897167	5.103587	5.173081333	4.447712532	4.971233035	5.112390877	5.22078623	5.139303948
4.153359	4.565328	5.283764	4.667483667	4.410421333	5.283764	4.667483667	5.144768796	4.971233035	5.112390877	5.22078623	3.902547582
5.191911	4.482289	5.07797	4.91739	5.0546505	5.07797	4.91739	4.566832984	4.971233035	5.112390877	5.22078623	4.212462488
3.806668	4.020909	5.541407	4.456328	4.131498	5.541407	4.456328	5.360579674	4.971233035	5.112390877	5.22078623	4.035250943
5.91171	6.822834	4.899645	5.878063	5.8948865	4.899645	5.878063	4.076670122	4.971233035	5.112390877	5.22078623	5.730540508
4.763376	2.985271	3.383203	3.710616667	4.236996333	3.383203	3.710616667	3.305152634	4.971233035	5.112390877	5.22078623	3.597253814
5.828828	4.141981	5.292341	5.087716667	5.458272333	5.292341	5.087716667	4.521801163	4.971233035	5.112390877	5.22078623	4.373923206
4.823521	5.852225	5.693756	5.456500667	5.140010833	5.693756	5.456500667	5.094838436	4.971233035	5.112390877	5.22078623	4.78128003
4.901828	4.245094	4.67986	4.608927333	4.755377667	4.67986	4.608927333	4.424728585	4.971233035	5.112390877	5.22078623	3.627538974
6.534007	4.337895	6.423141	5.765014333	6.149510667	6.423141	5.765014333	4.956975731	4.971233035	5.112390877	5.22078623	5.538876395
6.624266	3.889244	5.390714	5.301408	5.962837	5.390714	5.301408	3.992765184	4.971233035	5.112390877	5.22078623	5.201727707
5.845819	5.827471	4.531777	5.401689	5.623754	4.531777	5.401689	3.207078158	4.971233035	5.112390877	5.22078623	4.800731133
5.474306	4.245077	3.312901	4.344094667	4.909200333	3.312901	4.344094667	3.201928866	4.971233035	5.112390877	5.22078623	3.438455484
4.388281	4.210381	5.562507	4.720389667	4.554335333	5.562507	4.720389667	5.368162887	4.971233035	5.112390877	5.22078623	4.187019807
5.429981	4.276572	4.269004	4.658519	5.04425	4.269004	4.658519	2.795232181	4.971233035	5.112390877	5.22078623	4.507852514
4.182783	5.702815	4.985842	4.957146667	4.569964833	4.985842	4.957146667	3.67609146	4.971233035	5.112390877	5.22078623	4.555766527
6.191727	3.32326	2.309483	3.94149	5.0666085	2.309483	3.94149	2.289609765	4.971233035	5.112390877	5.22078623	3.754237357
7.340281	6.166309	2.666247	5.390945667	6.365613333	2.666247	5.390945667	2.520641832	4.971233035	5.112390877	5.22078623	5.34219972
4.783173	6.046561	5.281741	5.370491667	5.076832333	5.281741	5.370491667	4.830932783	4.971233035	5.112390877	5.22078623	5.292057644
4.424859	4.689727	4.578742	4.564442667	4.494650833	4.578742	4.564442667	4.258234969	4.971233035	5.112390877	5.22078623	4.527454871
7.749881	6.008586	4.253907	6.004124667	6.877002833	4.253907	6.004124667	2.950488391	4.971233035	5.112390877	5.22078623	5.680688111
7.411178	6.683396	4.334621	6.143065	6.7771215	4.334621	6.143065	4.120036333	4.971233035	5.112390877	5.22078623	5.655011148
7.725089	6.209489	4.06349	5.999356	6.8622225	4.06349	5.999356	3.192273949	4.971233035	5.112390877	5.22078623	5.513030258
4.098049	6.063624	3.713837	4.62517	4.3616095	3.713837	4.62517	2.645722927	4.971233035	5.112390877	5.22078623	4.507626725
5.638004	4.938797	5.641523	5.406108	5.522056	5.641523	5.406108	5.359227301	4.971233035	5.112390877	5.22078623	4.652189599
2.261085	7.56727	4.174903	4.667752667	3.464418833	4.174903	4.667752667	2.991661783	4.971233035	5.112390877	5.22078623	4.269957098
4.739951	5.920966	4.13286	4.931259	4.835605	4.13286	4.931259	3.630353899	4.971233035	5.112390877	5.22078623	4.88030434
4.48649	5.783055	4.679288	4.982944333	4.734717167	4.679288	4.982944333	3.795287462	4.971233035	5.112390877	5.22078623	4.050686908
5.106262	2.87988	4.260907	4.082349667	4.594305833	4.260907	4.082349667	3.230058657	4.971233035	5.112390877	5.22078623	3.870975268
5.294058	3.442415	4.944924	4.560465667	4.927261833	4.944924	4.560465667	3.911200813	4.971233035	5.112390877	5.22078623	3.8560873
4.540502	4.325219	9.057352	5.974357667	5.257429833	9.057352	5.974357667	7.600214523	4.971233035	5.112390877	5.22078623	5.575994823
3.974602	4.790526	4.620093	4.461740333	4.218171167	4.620093	4.461740333	4.103038383	4.971233035	5.112390877	5.22078623	3.897979529
5.57129	6.376151	6.997416	6.314952333	5.943121167	6.997416	6.314952333	6.22639231	4.971233035	5.112390877	5.22078623	5.877385333
4.099452	2.310166	5.280023	3.896547	3.9979995	5.280023	3.896547	5.142810987	4.971233035	5.112390877	5.22078623	3.470210637
3.540908	7.135742	5.236984	5.304544667	4.422726333	5.236984	5.304544667	3.870110333	4.971233035	5.112390877	5.22078623	4.986804391
5.862522	5.468016	7.681953	6.337497	6.1000095	7.681953	6.337497	7.414034907	4.971233035	5.112390877	5.22078623	5.46477616
3.398433	6.825256	4.511027	4.911572	4.1550025	4.511027	4.911572	4.499442866	4.971233035	5.112390877	5.22078623	4.746513579
4.951761	3.419356	4.453616	4.274911	4.613336	4.453616	4.274911	4.414968527	4.971233035	5.112390877	5.22078623	3.833065878
3.429133	3.844412	6.047054	4.440199667	3.934666333	6.047054	4.440199667	5.480195153	4.971233035	5.112390877	5.22078623	3.519848091
5.134808	3.881292	4.129734	4.381944667	4.758376333	4.129734	4.381944667	3.587226349	4.971233035	5.112390877	5.22078623	3.706960995
4.288021	3.249454	3.223976	3.587150333	3.937585667	3.223976	3.587150333	1.787268754	4.971233035	5.112390877	5.22078623	2.805027437
5.888044	2.889265	5.429319	4.735542667	5.311793333	5.429319	4.735542667	3.972929633	4.971233035	5.112390877	5.22078623	3.828423956
4.788309	5.147643	4.329253	4.755068333	4.771688667	4.329253	4.755068333	2.976635143	4.971233035	5.112390877	5.22078623	3.902607642
4.715687	7.177859	4.26067	5.384738667	5.050212833	4.26067	5.384738667	2.831899155	4.971233035	5.112390877	5.22078623	5.01118426
4.624745	7.194517	5.131934	5.650398667	5.137571833	5.131934	5.650398667	3.926898639	4.971233035	5.112390877	5.22078623	4.901082511
6.943164	4.347986	3.497123	4.929424333	5.936294167	3.497123	4.929424333	3.464209357	4.971233035	5.112390877	5.22078623	4.173372309
4.98238	5.255177	7.038943	5.758833333	5.370606667	7.038943	5.758833333	6.216407213	4.971233035	5.112390877	5.22078623	4.845888105
6.785792	7.407878	5.563556	6.585742	6.685767	5.563556	6.585742	4.762008497	4.971233035	5.112390877	5.22078623	5.923882608
3.321292	4.616851	3.226516	3.721553	3.5214225	3.226516	3.721553	2.074927881	4.971233035	5.112390877	5.22078623	3.077002872
7.411178	6.683396	4.334621	6.143065	6.7771215	4.334621	6.143065	4.120036333	4.971233035	5.112390877	5.22078623	5.655011148
7.725089	6.209489	4.06349	5.999356	6.8622225	4.06349	5.999356	3.192273949	4.971233035	5.112390877	5.22078623	5.513030258
4.098049	6.063624	3.713837	4.62517	4.3616095	3.713837	4.62517	2.645722927	4.971233035	5.112390877	5.22078623	4.507626725
5.638004	4.938797	5.641523	5.406108	5.522056	5.641523	5.406108	5.359227301	4.971233035	5.112390877	5.22078623	4.652189599
2.261085	7.56727	4.174903	4.667752667	3.464418833	4.174903	4.667752667	2.991661783	4.971233035	5.112390877	5.22078623	4.269957098
4.739951	5.920966	4.13286	4.931259	4.835605	4.13286	4.931259	3.630353899	4.971233035	5.112390877	5.22078623	4.88030434
4.48649	5.783055	4.679288	4.982944333	4.734717167	4.679288	4.982944333	3.795287462	4.971233035	5.112390877	5.22078623	4.050686908
5.106262	2.87988	4.260907	4.082349667	4.594305833	4.260907	4.082349667	3.230058657	4.971233035	5.112390877	5.22078623	3.870975268
5.294058	3.442415	4.944924	4.560465667	4.927261833	4.944924	4.560465667	3.911200813	4.971233035	5.112390877	5.22078623	3.8560873
4.540502	4.325219	9.057352	5.974357667	5.257429833	9.057352	5.974357667	7.600214523	4.971233035	5.112390877	5.22078623	5.575994823
3.974602	4.790526	4.620093	4.461740333	4.218171167	4.620093	4.461740333	4.103038383	4.971233035	5.112390877	5.22078623	3.897979529
5.57129	6.376151	6.997416	6.314952333	5.943121167	6.997416	6.314952333	6.22639231	4.971233035	5.112390877	5.22078623	5.877385333
4.099452	2.310166	5.280023	3.896547	3.9979995	5.280023	3.896547	5.142810987	4.971233035	5.112390877	5.22078623	3.470210637
3.540908	7.135742	5.236984	5.304544667	4.422726333	5.236984	5.304544667	3.870110333	4.971233035	5.112390877	5.22078623	4.986804391
5.862522	5.468016	7.681953	6.337497	6.1000095	7.681953	6.337497	7.414034907	4.971233035	5.112390877	5.22078623	5.46477616
3.398433	6.825256	4.511027	4.911572	4.1550025	4.511027	4.911572	4.499442866	4.971233035	5.112390877	5.22078623	4.746513579
4.951761	3.419356	4.453616	4.274911	4.613336	4.453616	4.274911	4.414968527	4.971233035	5.112390877	5.22078623	3.833065878
3.429133	3.844412	6.047054	4.440199667	3.934666333	6.047054	4.440199667	5.480195153	4.971233035	5.112390877	5.22078623	3.519848091
5.134808	3.881292	4.129734	4.381944667	4.758376333	4.129734	4.381944667	3.587226349	4.971233035	5.112390877	5.22078623	3.706960995
4.288021	3.249454	3.223976	3.587150333	3.937585667	3.223976	3.587150333	1.787268754	4.971233035	5.112390877	5.22078623	2.805027437
5.888044	2.889265	5.429319	4.735542667	5.311793333	5.429319	4.735542667	3.972929633	4.971233035	5.112390877	5.22078623	3.828423956
4.788309	5.147643	4.329253	4.755068333	4.771688667	4.329253	4.755068333	2.976635143	4.971233035	5.112390877	5.22078623	3.902607642
4.715687	7.177859	4.26067	5.384738667	5.050212833	4.26067	5.384738667	2.831899155	4.971233035	5.112390877	5.22078623	5.01118426
4.624745	7.194517	5.131934	5.650398667	5.137571833	5.131934	5.650398667	3.926898639	4.971233035	5.112390877	5.22078623	4.901082511
6.943164	4.347986	3.497123	4.929424333	5.936294167	3.497123	4.929424333	3.464209357	4.971233035	5.112390877	5.22078623	4.173372309
4.98238	5.255177	7.038943	5.758833333	5.370606667	7.038943	5.758833333	6.216407213	4.971233035	5.112390877	5.22078623	4.845888105
5.134808	3.881292	4.129734	4.381944667	4.758376333	4.129734	4.381944667	3.587226349	4.971233035	5.112390877	5.22078623	3.706960995
4.288021	3.249454	3.223976	3.587150333	3.937585667	3.223976	3.587150333	1.787268754	4.971233035	5.112390877	5.22078623	2.805027437
5.888044	2.889265	5.429319	4.735542667	5.311793333	5.429319	4.735542667	3.972929633	4.971233035	5.112390877	5.22078623	3.828423956
4.788309	5.147643	4.329253	4.755068333	4.771688667	4.329253	4.755068333	2.976635143	4.971233035	5.112390877	5.22078623	3.902607642
4.715687	7.177859	4.26067	5.384738667	5.050212833	4.26067	5.384738667	2.831899155	4.971233035	5.112390877	5.22078623	5.01118426
4.624745	7.194517	5.131934	5.650398667	5.137571833	5.131934	5.650398667	3.926898639	4.971233035	5.112390877	5.22078623	4.901082511
6.943164	4.347986	3.497123	4.929424333	5.936294167	3.497123	4.929424333	3.464209357	4.971233035	5.112390877	5.22078623	4.173372309
3.429133	3.844412	6.047054	4.440199667	3.934666333	6.047054	4.440199667	5.480195153	4.971233035	5.112390877	5.22078623	3.519848091
5.134808	3.881292	4.129734	4.381944667	4.758376333	4.129734	4.381944667	3.587226349	4.971233035	5.112390877	5.22078623	3.706960995
4.288021	3.249454	3.223976	3.587150333	3.937585667	3.223976	3.587150333	1.787268754	4.971233035	5.112390877	5.22078623	2.805027437
5.888044	2.889265	5.429319	4.735542667	5.311793333	5.429319	4.735542667	3.972929633	4.971233035	5.112390877	5.22078623	3.828423956
4.788309	5.147643	4.329253	4.755068333	4.771688667	4.329253	4.755068333	2.976635143	4.971233035	5.112390877	5.22078623	3.902607642
4.715687	7.177859	4.26067	5.384738667	5.050212833	4.26067	5.384738667	2.831899155	4.971233035	5.112390877	5.22078623	5.01118426
4.624745	7.194517	5.131934	5.650398667	5.137571833	5.131934	5.650398667	3.926898639	4.971233035	5.112390877	5.22078623	4.901082511
3.429133	3.844412	6.047054	4.440199667	3.934666333	6.047054	4.440199667	5.480195153	4.971233035	5.112390877	5.22078623	3.519848091
5.134808	3.881292	4.129734	4.381944667	4.758376333	4.129734	4.381944667	3.587226349	4.971233035	5.112390877	5.22078623	3.706960995
4.288021	3.249454	3.223976	3.587150333	3.937585667	3.223976	3.587150333	1.787268754	4.971233035	5.112390877	5.22078623	2.805027437
5.888044	2.889265	5.429319	4.735542667	5.311793333	5.429319	4.735542667	3.972929633	4.971233035	5.112390877	5.22078623	3.828423956
4.788309	5.147643	4.329253	4.755068333	4.771688667	4.329253	4.755068333	2.976635143	4.971233035	5.112390877	5.22078623	3.902607642
3.429133	3.844412	6.047054	4.440199667	3.934666333	6.047054	4.440199667	5.480195153	4.971233035	5.112390877	5.22078623	3.519848091
5.134808	3.881292	4.129734	4.381944667	4.758376333	4.129734	4.381944667	3.587226349	4.971233035	5.112390877	5.22078623	3.706960995
4.288021	3.249454	3.223976	3.587150333	3.937585667	3.223976	3.587150333	1.787268754	4.971233035	5.112390877	5.22078623	2.805027437
5.888044	2.889265	5.429319	4.735542667	5.311793333	5.429319	4.735542667	3.972929633	4.971233035	5.112390877	5.22078623	3.828423956
4.788309	5.147643	4.329253	4.755068333	4.771688667	4.329253	4.755068333	2.976635143	4.971233035	5.112390877	5.22078623	3.902607642
4.715687	7.177859	4.26067	5.384738667	5.050212833	4.26067	5.384738667	2.831899155	4.971233035	5.112390877	5.22078623	5.01118426
4.624745	7.194517	5.131934	5.650398667	5.137571833	5.131934	5.650398667	3.926898639	4.971233035	5.112390877	5.22078623	4.901082511
6.943164	4.347986	3.497123	4.929424333	5.936294167	3.497123	4.929424333	3.464209357	4.971233035	5.112390877	5.22078623	4.173372309
4.98238	5.255177	7.038943	5.758833333	5.370606667	7.038943	5.758833333	6.216407213	4.971233035	5.112390877	5.22078623	4.845888105
5.134808	3.881292	4.129734	4.381944667	4.758376333	4.129734	4.381944667	3.587226349	4.971233035	5.112390877	5.22078623	3.706960995
4.288021	3.249454	3.223976	3.587150333	3.937585667	3.223976	3.587150333	1.787268754	4.971233035	5.112390877	5.22078623	2.805027437
5.888044	2.889265	5.429319	4.735542667	5.311793333	5.429319	4.735542667	3.972929633	4.971233035	5.112390877	5.22078623	3.828423956
4.788309	5.147643	4.329253	4.755068333	4.771688667	4.329253	4.755068333	2.976635143	4.971233035	5.112390877	5.22078623	3.902607642
4.715687	7.177859	4.26067	5.384738667	5.050212833	4.26067	5.384738667	2.831899155	4.971233035	5.112390877	5.22078623	5.01118426

## Conclusion

This article aimed to introduce CEV CDEC and CM CDEC charts as tools for monitoring type-I censored data, employing CEV and CM methodologies. The effectiveness of the suggested control charts was assessed under a range of scenarios, including various sorts of upward and downward shifts, varied subgroup sizes, rates of censoring, and selections of parameters. The performance of the suggested charts was also evaluated in comparison to the CM-DE chart and CM CDE chart. The Weibull distribution was chosen as an example to demonstrate the suggested methodology because of its practical significance in reliability, life testing, and medical research. However, any other suitable lifetime distribution might also be utilized. Additionally, the performance of the censored data charts was evaluated under the estimated parameter case. The article used ARL as a performance criterion to evaluate the efficiency of the proposed control charts.

The ARL analysis showed that the CM and CEV based CDEC charts outperformed the CM-DE and CM CDE charts, with the CM approach being more effective than the CEV approach. This can be explained by the fact that the conditional median is less sensitive to extreme observations compared to the conditional mean, resulting in fewer false alarms. The ARL values decreased with increasing censoring rates, but increased with increasing shape parameter values, indicating improved chart efficiency. The performance of the censored charts was found to be compromised in the estimated parameter case compared to the known parameter case, highlighting the need for large Phase-I data sets to minimize estimation effects on chart performance, as recommended in the literature.

The cancer data on patients recovery time is monitored using the proposed control charting methodology. In future studies, non parametric control charts can be explored, and the impact of simultaneous estimation of shape and scale parameters can be investigated.

## Data Availability

The data used in the article are available from the corresponding author. Also, the reference of real data is cited in the text.

## Abbreviations

CEV: Conditional expected value
CM: Conditional median
EWMA: Exponentially weighted moving average
DE: Dual EWMA
CDE: Composite dual EWMA
CUSUM: Cumulative sum
CDEC: Composite dual exponentially weighted moving average cumulative sum
ARL: Average run length
SPC: Statistical process control

## Code availability

An algorithm is mentioned to replicate the results.
